# GIT1 regulates angiogenic factor secretion in bone marrow mesenchymal stem cells via NF‐κB/Notch signalling to promote angiogenesis

**DOI:** 10.1111/cpr.12689

**Published:** 2019-09-10

**Authors:** Linwei Li, Pengyu Tang, Zheng Zhou, Qian Wang, Tao Xu, Shujie Zhao, Yifan Huang, Fanqi Kong, Wei Liu, Lin Cheng, Zhimin Zhou, Xuan Zhao, Changjiang Gu, Yongjun Luo, Gaojian Tao, Dingfei Qian, Jian Chen, Jin Fan, Guoyong Yin

**Affiliations:** ^1^ Department of Orthopedic The First Affiliated Hospital of Nanjing Medical University Nanjing China; ^2^ Department of Pain Affiliated Drum Tower Hospital Medical School of Nanjing University Nanjing China

**Keywords:** angiogenesis, fracture healing, GIT1, NF‐κB, Notch

## Abstract

**Objectives:**

Osteogenesis is coupled with angiogenesis during bone remodelling. G‐protein‐coupled receptor (GPCR) kinase 2‐interacting protein‐1 (GIT1) is an important protein that participates in fracture healing by regulating angiogenesis. This study investigated whether GIT1 could affect bone mesenchymal stem cells (BMSCs) to secrete angiogenic factors to enhance fracture healing by promoting angiogenesis and its possible mechanism.

**Materials and methods:**

The angiogenesis of mice post‐fracture was detected by micro‐CT and immunofluorescence. Subsequently, vascular endothelial growth factor (VEGF) level in mouse and human BMSCs (hBMSCs) under TNF‐α stimulation was detected. The hBMSCs were transfected with GIT1 shRNAs to further explore the relationship between GIT1 and VEGF and angiogenesis in vitro. Furthermore, based on previous research on GIT1, possible signal pathways were investigated.

**Results:**

GIT1 knockout mice exhibited impaired angiogenesis and delayed fracture healing. And GIT1 deficiency remarkably reduced the expression of VEGF mRNA in BMSCs, which affected the proliferation and migration of human umbilical vein endothelial cells. GIT1 knockdown inhibited the activation of Notch and NF‐κB signals by decreasing nuclear transportation of NICD and P65/P50, respectively. Overexpression of the canonical NF‐κB subunits P65 and P50 markedly increased NICD‐dependent activation of recombination signal‐binding protein‐jκ reporter. Finally, GIT1 enhanced the affinity of NF‐κB essential modulator (NEMO) for K63‐linked ubiquitin chains via interaction with NEMO coiled‐coil 2 domains.

**Conclusion:**

These data revealed a positive role for GIT1 by modulating the Notch/NF‐κB signals which promoting paracrine of BMSCs to enhance angiogenesis and fracture healing.

**Highlights**

- GIT1 knockdown impairs angiogenesis in fracture callus, possibly due to decreased VEGF secretion in BMSCs during early fracture.
- GIT1 deletion inhibits activation of Notch and canonical NF‐κB signals.
- GIT1 specificity enhances affinity between NEMO and K63‐linked ubiquitin chains via interactions of GIT1 and NEMO CC2 domains.
- GIT1 does not affect K63‐linked ubiquitination of TNF RIP1 mediated by TRAF2.

## 1 INTRODUCTION

Initial haematoma formation after fracture is followed by inflammation, repair, and finally, remodelling. The inflammatory phase is a critical period characterized by impaired perfusion and migration of a wide array of osteoprogenitor cells, bone mesenchymal cells (BMSCs) and osteoblast cells to the site of injury^1^, ^2^ for further release of inflammatory cytokines within 3‐7 days of injury.^3^ Therefore, activation of the NF‐κB signal via inflammatory factors, such as TNF‐α, IL‐1β and IL‐6, is involved in the regulation of fracture healing.^3^, ^4^, ^5^, ^6^ During this stage, TNF‐α, synergistically with IL‐1β, initiate the bone healing cascade and push it towards endochondral bone formation, promoting matrix mineralization by BMSCs in vitro, which is essential for murine bone regeneration in vivo.^7^, ^8^, ^9^ These inflammatory factors can also induce BMSCs to produce a variety of angiogenic factors that are involved in the regulation of angiogenesis in the early stages of the healing process.^10^, ^11^ Of these factors, vascular endothelial growth factor (VEGF) is particularly important in angiogenesis, which is in turn critical for the VEGF‐dependent pathway related to bone formation.^10^, ^11^, ^12^, ^13^, ^14^, ^15^, ^16^ Consequently, bone fracture or injury initiates a series of cellular and molecular pathways that commence with a haematoma formation and an inflammatory cascade that regulates BMSC activity and paracrine effects, leading to fracture healing and reestablishment of skeletal integrity.^5^

NF‐κB is a family of transcription factors that regulate many aspects of normal cellular functions, as well as innate and adaptive immunity in response to pathogens and autoimmune stimuli.^17^, ^18^ The family includes NF‐κB1 (also known as P50 and its precursor P105), NF‐κB2 (P52 and its precursor p100), RELA (P65), RELB and c‐REL. Homo‐ and heterodimers of these proteins activate transcription of target genes, typically through canonical (P65/P50) and non‐canonical (RELB/P52) signalling.^19^, ^20^ Importantly, NF‐κB essential modulator (NEMO), also known as IKKγ, interacts with the ubiquitin chains and is considered to be the key activator of the canonical NF‐κB signal.^21^, ^22^, ^23^, ^24^, ^25^

Notch is a family of evolutionarily conserved receptors that regulate cell fate and VEGF expression in a variety of cells, including BMSCs.^26^, ^27^ Notch receptors are activated following direct contact with their ligands expressed on adjacent cells. Notch receptors have extracellular, transmembrane and intracellular domains. Upon ligand binding, Notch intracellular domain (NICD) of the receptor is cleaved by γ‐secretase and translocates to the nucleus, where it associates with the recombination signal‐binding protein‐jκ (RBP‐jк), leading to the transcriptional activation of target genes, such as Hey1 and Hes1. Notch activation depends upon crosstalk with other regulatory pathways, including NF‐κB.^20^, ^28^, ^29^, ^30^, ^31^ We speculated that the NF‐κB and Notch signals regulate secretion of angiogenic factors in BMSCs during the inflammatory phase of fracture healing.

G‐protein‐coupled receptor (GPCR) kinase 2‐interacting protein‐1 (GIT1) binds to the G‐protein‐coupled receptor kinase 2 (GRK2) and is involved in the endocytosis of adrenergic receptors.^32^, ^33^ GIT1 interacts with several signalling molecules via its functional domains and possesses diverse physiological functions.^34^ Our previous studies have suggested that GIT1 also plays a crucial role in fracture healing by regulating the function of osteoclasts under normal conditions,^35^ migration of osteoblasts^36^ and number of osteoclasts^37^, ^38^ during fracture healing and impaired angiogenesis.^38^, ^39^

To the best of our knowledge, the detailed mechanism by which GIT1 affects secretion and expression of angiogenic factors, particularly VEGF during fracture healing, remains unknown. Here, we discovered that GIT1 knockout (GIT1 KO) mice had impaired angiogenesis in fracture callus tissue, resulting in delayed fracture healing. The expression of VEGF mRNA in BMSCs from bone marrow adjacent to the fracture site by adherent culture was reduced in GIT1 KO mice, compared to that in control littermates 3‐7 days post‐fracture. In vitro experiments further confirmed that GIT1 deficiency could reduce VEGF secretion and expression, consistent with the activated state of NF‐κB and Notch signals in BMSCs. Further studies suggested that the combination between GIT1 and NEMO specifically promoted the affinity for K63‐linked polyubiquitin, thereby activating the canonical NF‐κB and Notch signals, also known as NF‐κB cross‐linking signals, which ultimately regulate the expression of angiogenic factors.

## 2 MATERIALS AND METHODS

### 2.1 Reagents and antibodies

Cytokine TNF‐α (Peprotech), antagonist DAPT (MCE) and MG132 (MCE) were used to treat cells. Lipofectamine 2000 (Thermo Fisher Scientific) and protein A/G magnetic beads (Pierce Biotechnology) were used for cell transfection and co‐immunoprecipitation (co‐IP) analysis. Antibodies included the following: mouse anti‐GIT1 (Novus); rabbit anti‐NEMO, p‐IKKα/β, NICD, RelB, P65, p‐P65, P52, P50, GAPDH and GAPDH (CST); rabbit anti‐IKKα, IKKβ, RIP1, Hey1, Hes1 and CD31 (Abcam); mouse anti‐TRAF2 and EMCN (Santa Cruz); mouse anti‐His, HA, Flag and Myc (MultiScience); horseradish peroxidase‐conjugated goat anti‐rabbit IgG (H+L) and horseradish peroxidase‐conjugated goat anti‐mouse IgG (H+L) (Invitrogen); Alexa Fluor 488‐ or 594‐conjugated goat anti‐mouse IgG (H+L) (Jackson); and Alexa Fluor 488‐ or Alexa Fluor 594‐conjugated goat anti‐rabbit IgG (H+L) (Jackson). Nuclei were stained with DAPI dihydrochloride (Thermo Fisher Scientific).

### 2.2 Stabilized fracture model

All animal protocols were approved by the Animal Committee at the First Affiliated Hospital of Nanjing Medical University. Stabilized femur fractures were produced in 8‐week GIT1 KO mice (C57BL/6 background, a gift from Bradford C. Berk, University of Rochester, Cardiovascular Research Institute) and control mice with C57BL/6 background, as described previously.^34^, ^35^, ^37^, ^38^, ^40^

### 2.3 Micro‐CT system

Micro‐CT system (SkyScan 1172; Bruker) was used to assess callus volume and vascularity. Scanning parameters were as follows: 18‐μm resolution, 0.2‐nm aluminium filter, 80‐kV voltage and 112‐μA current. Vascular networks at the cortical bone junction and around the fractures were examined using micro‐CT analysis combined with contrast agent perfusion. Briefly, blood vessels were first rinsed with normal saline containing heparin and 4% PFA. Then, using MICROFIL® injection compound (Flow Tech, Inc) contrast media, a radiopaque silicone rubber compound containing lead chromate was perfused via the heart. After perfusion, the fractured femur was removed and scanned using a micro‐CT system. The samples were subsequently decalcified for 10 days using a 10% EDTA solution. After complete decalcification, the samples were scanned again to visualize only the vascularization within the callus tissue. 3D reconstructions were made using NRecon software (ver. 1.6.9.4; Bruker).

### 2.4 Immunohistochemistry

For histology analysis, femurs were isolated from mice after perfusion with 4% PFA and fixed in 4% PFA overnight at room temperature, followed by decalcification in 14% EDTA for 2 weeks. After decalcification, femurs were paraffin‐embedded and then sectioned into 6‐μm thick slices. Haematoxylin and eosin (H&E) and CD31/ EMCN double immunofluorescent staining was performed on paraffin sections according to standard procedure.

### 2.5 Plasmid production

Full‐length sequences for human GIT1, NEMO and ubiquitin were subcloned into the EcoRI and NotI sites of the Flag‐, HA‐ and myc‐tagged pcDNA3.1 vectors (Thermo Fisher Scientific). RT‐PCR was used to clone cDNAs for GIT1 (1‐250 aa), GIT1 (1‐420 aa), GIT1 (1‐620 aa), GIT1 (250‐770 aa), GIT1 (620‐770 aa), GIT1 ΔCC2 (lacking synaptic localization domain containing the CC2 domain present in aa 421‐619), NEMO (1‐250 aa), NEMO (250‐419 aa) and NEMO ΔCC2 (lacking the CC2 domain present in aa 250‐300) into corresponding vectors. Ubiquitin combination mutants were generated using PCR or the QuikChange Multi Site‐Directed Mutagenesis Kit (200515, Agilent Technologies UK Ltd.).

### 2.6 Luciferase assay

A total of 5 × 10^4^ HEK293T cells with and without GIT1 deletion were seeded in 96‐well plates and co‐transfected with NOTCH1‐NICD (0.05 μg), RELB‐, P65‐, P52‐ and/or P50‐expressing constructs (GenePharma), or corresponding empty vectors along with RBP‐jκ‐Luc (0.5 μg) and pRL‐renilla (0.01 μg; Promega). Cells were cultured for a further 48 hours followed by harvesting for dual luciferase activity assays (Promega), according to the manufacturer's instructions. RBP‐jκ‐Luc reporter activity was defined as the ratio of Firefly/Renilla luciferase activities.

### 2.7 Cell culture and transfection

hBMSCs (PCS‐500‐012™) were purchased from ATCC. For the experiments, hBMSCs were grown in culture medium, consisting of MSCM (ScienCell) enriched with 5% FBS (No. 0025), 1% mesenchymal stem cell growth supplement MSCGS (No. 7552) and 1% penicillin/streptomycin solution (No. 0503) in a humidified atmosphere of 5% CO_2_ at 37°C. At 80% confluence, cells were detached and seeded (10 000 cells/cm^2^) <10 times. hBMSCs were infected with lentiviral GV112 vector carrying a target gene sequence or a scrambled shRNA 5 days after the in vitro culture, according to the manufacturer's instructions. The cells were processed for subsequent experiments 48 hours after transfection. HEK293T cells and HUVECs were maintained at 5% CO_2_ and 37°C in DMEM (Hyclone) supplemented with 10% FBS (Hyclone). HEK293T cells were transfected with expression vectors for the indicated proteins using Lipofectamine 2000 (Invitrogen). Overexpression efficiency was detected by Western blotting.

### 2.8 Primary mBMSC isolation and flow cytometry analysis

GIT1 wild‐type (WT) and KO mice between 0 and 7 days post‐fracture were anesthetized with 10% chloral hydrate (1 mL/300 g, i.p.) and then disinfected the lower limbs of the mice with 75% ethanol. Under aseptic conditions, the bilateral lower limb femurs of mice were obtained, followed by removal of attached fatty and connective tissues. The femurs were stored in sterile culture dishes. After washing with PBS, the distal fracture region was resected. The femur marrow cavities were rinsed with MSCM three to four times. The rinsing fluid was collected in 50‐mL tubes, and mixing mixed cells of fluid with moderate medium. After the rinsing fluid was filtered with 70‐μm cell strainer (CORNING), it was centrifuged (270 *g*, 5 minutes) and resuspended. The cells at an adjusted density of 1 × 10^6^ cells/mL were inoculated in a‐25 cm^2^ culture flask and cultured at 37°C and 5% CO_2_ with saturated humidity. After 24 hours, non‐adherent cells were discarded and adherent cells were cultured further. The medium was changed every 3 days. After the adherent cells reached 80%‐90% confluency, culture medium was removed and the cells were washed with PBS three times and subsequently digested with 0.25% EDTA‐trypsin. The primary mouse BMSCs (mBMSCs) were then prepared for VEGF mRNA qPCR analysis. After several cell passages, single‐cell suspensions were prepared from P3‐P5 mBMSCs. After fixing in 4% PFA for 15 minutes, the cells were blocked with 5% normal goat serum for 1 hour at 4°C and incubated with fluorescein‐labelled antibodies (eBioscience), including anti‐CD44 (PE), anti‐CD45 (PE), anti‐CD90 (PE) and anti‐CD105 (PE). The non‐specific mouse IgG served as an isotype control. A total of 5 × 10^5^ labelled cells were evaluated and fluorescence signals were subsequently determined using a flow cytometer (FACSCalibur, BD Biosciences).

### 2.9 Endothelial tube formation assay

After thawing on ice, a total of 100 μL of Matrigel (CORNING) were plated in 96‐well plates and incubated at 37°C for 30 minutes to allow the Matrigel to polymerize. HUVECs (10,000 cells/100 μL medium/well) were then added to each well. When the cells became adherent, the medium was replaced with conditioned medium (CM) from the hBMSC supernatant, which was treated or untreated with GIT1‐shRNA. Some HUVECs were then treated with the VEGF antibody. The plates were then incubated for 8 hours at 37°C and 5% CO_2_ in the humidified atmosphere. At the end of incubation, each well was photographed with a digital camera (Nikon Inc) and total tube length and total branch points in each chamber were carefully measured.

### 2.10 Migration assay

Transwell assay was used to analyse the effect of GIT1 KO in hBMSCs on HUVEC migration ability. Briefly, HUVECs (20 000 cells/chamber) were seeded into the upper chamber of a 24‐well Transwell plate (Corning; pore size: 8 µm) with serum‐free DMEM and 600 µL/well of hBMSC‐CM treated or untreated with VEGF antibody were added to the lower chamber. After co‐incubation for 48 hours in 37°C in 5% CO_2_, cells from the upper surface of the filter membranes were wiped away with a cotton swab. Cells that migrated to the lower surface of the filter membrane were stained with 0.5% crystal violet for 1 hour. Migratory activity was assessed by observing stained HUVECs under a microscope. Migrated cell numbers and migration rates were then measured.

### 2.11 Wound‐healing assay

In vitro wound‐healing assay was performed using the ibidi Culture‐Inserts (GMBH), according to the manufacturer's instructions. An ibidi Culture‐Insert consisting of two wells separated by a 500‐μm thick wall was placed into the wells of a 6‐well plate and slightly pressed on top with tweezers to ensure a tight adhesion. An equal number of HUVECs (20 000 cells/100 μL medium/well) were added into the two wells of the same insert with serum‐free DMEM. After 6 hours, the insert was gently removed, creating a gap of 500 μm, and culture medium was replaced with conditional medium. The cells were then treated with the VEGF antibody and migration was observed and photographed after 0 and 12 hours using a digital microscope (Nikon Inc). The per cent of cellular migration after scratches made was determined as analysis of change in pixels due to migration front, as measured using Image J software.

### 2.12 Cell viability assay

Cell Counting Kit‐8 (CCK8, Dojindo) was used for cell viability assays. Briefly, HUVECs were seeded in a 96‐well plate at a density of 2000 cells/100 μL medium/well. When cells attached to the wall, the culture medium was replaced with CM with or without the VEGF antibody. Cell viabilities were determined at 0, 12, 24 and 48 hours. A total of 10 μL of CCK8 were added to each well and incubated for 2 hours at 37°C. Optical density values at 450‐nm wavelength were determined by a microplate reader (ELx800, Bio‐Tek).

### 2.13 Lentiviral vector production

Human GIT1 shRNA target sequences were as follows: shRNA 1:5′‐GATCACAAGAATGGGCATT‐3′, shRNA 2:5′‐CACCTTGATCATCGACATT‐3′ and shRNA 3:5′‐TGCTCAGAGAAGATCCATT‐3′. An additional scrambled sequence (5′‐TTCTCCGAACGTGTCACGT‐3′) was also designed as a negative control (NC). Lentivirus containing human GIT1 shRNAs (sh‐GIT1 1, 2 and 3) or NC shRNA (sh‐Scr) was packaged using the GV112 vector.

### 2.14 Enzyme‐linked immunosorbent assay (ELISA)

To determine whether GIT1 KO will affect hBMSC Notch activity in each group, the levels of VEGF (R&D) in hBMSC supernatant were measured using an ELISA kit, according to the manufacturer's instructions. Absorbance was read at a 450‐nm wavelength using a microplate reader (ELx800, Bio‐Tek).

### 2.15 RNA isolation and reverse‐transcription PCR and real‐time reverse‐transcription PCR analysis

Total RNA was extracted from cells using the Trizol reagent (Invitrogen), according to the manufacturer's instructions. Concentrations of total RNA were measured using a Biometra Optical Thermocycler (Analytik Jena). RNA (500 ng) was converted into cDNA with the High‐Capacity cDNA Reverse Transcription Kit (Thermo Fisher Scientific), according to the manufacturer's instructions. The human primer sequences were as follows:

GIT1: 5′‐ATGGTGCACACGCTTGCCAGC‐3′ (forward) and 5′‐TGCCTGTCCGCACGCTCGAGT‐3′ (reverse);
Hey1: 5’‐CCACGCTCCGCCACCATGAA‐3′ (forward) and 5′‐CGGCGCTTCTCGATGATGCCT‐3′ (reverse);
Hes1: 5′‐AACCAAAGACAGCATCTGAGCAC‐3′ (forward) and 5′‐TGTAGACCATGTAGTTGAGGTCA‐3′ (reverse);
VEGF: 5′‐GCGGGAAATCGTGCGTGACATT‐3′ (forward) and 5′‐GATGGAGTTGAAGGTAGTTTCGTG‐3′ (reverse);
GAPDH: 5′‐TTGCCATCAATGACCCCTTCA‐3′ (forward) and 5′‐CGCCCCACTTGATTTTGGA‐3′ (reverse).
Ang‐1:5′‐TCGTGAAGATGGAAGTCTAG‐3′ (forward) and 5′‐TGCCACTTTATCCCATTCAG‐3′ (reverse).
FGF: 5′‐GACGGCAGAGTTGACGG‐3′ (forward) and 5′‐CTCTCTCTT‐CTGCTTGAAGTTGTAGC‐3′ (reverse).
HGF: 5′‐GATGGCCAGCCGAGGC‐3′ (forward) and 5′‐TCAGCCCATGTTTTAATTGCA‐3′ (reverse).
TGF‐β: 5′‐GCTGAGCGCTTTTCTGATCCT‐3′ (forward) and 5′‐CGAGTGTGCTGCAGGTAGACA‐3′ (reverse).

The mouse sequences were as follows:

GAPDH: 5′‐CTCTTGCTCTCAGTATCCTTG‐3′ (forward) and 5′‐ GCTCACTGGCATGGCCTTCC‐3′ (reverse);
VEGF: 5′‐ACATCTTCAAGCCGTCCTGTGTGC‐3′ (forward) and 5′‐AAATGGCGAATCCAGTCCCACGAG‐3′ (reverse);
Ang‐1:5′‐GACACCTTGAAGGAGGAGAAAG‐3′ (forward) and 5′‐GTGTCCATGAGCTCCAGTTGT ‐3′ (reverse);
FGF: 5′‐TGTCTATCAAGGGAGTGTGTGC‐3′ (forward) and 5′‐ CAACTGGAGTATTTCCGTGACC‐3′ (reverse);
HGF: 5′‐TCACACAGAATCAGGCAAGACT‐3′ (forward) and 5′‐ AAGGGGTGTCAGGGTCAA‐3′ (reverse);
TGF‐β: 5′‐CTCCCGTGGCTTCTAGTGC‐3′ (forward) and 5′‐ GCCTTAGTTTGGACAGGATCTG ‐3′ (reverse);

Quantitative real‐time PCR was performed using SYBR qRCR premix (Takara). Cycling conditions included an initial denaturation at 95°C for 30 seconds, followed by 40 cycles at 95°C for 5 seconds each, 60°C for 30 seconds each and 72°C for 10 minutes each. Data were normalized using the ∆∆*C*_T_ method.

### 2.16 Immunofluorescence staining

Tissues or cells were fixed with 4% PFA for 10 minutes, washed with PBS three times, permeabilized with 0.05% Triton X‐100 for 10 minutes and blocked with 10% normal goat serum for 1 hour. Cells were incubated with primary antibodies overnight at 4°C, followed by Alexa Fluor 488‐ and Alexa Flour 594‐conjugated goat secondary antibodies (Jackson) for 1 hour at room temperature. After triple washing by PBS, nuclei were stained with DAPI (Thermo Fisher Scientific) and fluorescent images were acquired using an epifluorescence microscope (AxioVertA1 and ImagerA2) or a confocal fluorescence microscope (LSM510; Carl Zeiss).

### 2.17 Western blotting

Proteins were extracted from cells and callus tissue using the RIPA lysis and extraction buffer (KeyGen Biotechnology). Protein concentration was determined using the Bradford method. Equal amounts of protein were separated by SDS‐PAGE, transferred to PVDF membranes (SEQ00010; EMD Millipore) and incubated overnight at 4°C with primary antibodies followed by blocking with bovine serum albumin (5%, v/v). Membranes were then incubated for 120 minutes at room temperature with the secondary antibody. Reacting bands were visualized using enhanced chemiluminescence reagents (Pierce Biotechnology) and protein band density was semi‐quantified using ImageJ (National Institutes of Health).

### 2.18 Co‐IP

After treatments, cells were rinsed once with ice‐cold PBS and lysed on ice for 20 minutes in 1 mL of ice‐cold buffer A (20 mM TrisHCl, pH 7.4, 150 mM NaCl, 1% Triton X‐100, 0.5% sodium deoxycholate, 12 mM glycerophosphate, 10 mM sodium fluoride, 5 mM EGTA, 2 mM sodium vanadate, 1 mM PMSF, 2 mg/mL aprotinin and 2 mg/mL leupeptin). Cell extracts were clarified by centrifugation and supernatants were immunoprecipitated with the indicated antibodies specific for co‐IP using protein A/G magnetic beads. To detect protein ubiquitination, protein was harvested after the MG132 (10 μM) treatment for 3 hours and then immunoprecipitated with antibodies. Beads were washed three times with buffer A and subjected to SDS‐PAGE, followed by immunoblotting analysis.

### 2.19 Proximity ligation assay (PLA)

Cells were cultured in cover glass slide chambers (Thermo Scientific, 155360). After fixation with 4% PFA, cells were subjected to PLA using a Duolink detection kit and Detection Reagents Red (Sigma‐Aldrich, DUO94004 [Detection Solution, DUO84004; Ligation Buffer, DUO82009; Amplification Buffer, DUO82050; Ligase; Polymerase]), according to the manufacturer's instructions with minor modifications. Briefly, permeabilized cells were blocked and incubated with primary antibodies overnight at 4°C. After incubation with secondary antibodies conjugated to unique DNA probes (anti‐mouse and anti‐rabbit for two primary antibodies provided by the kit), a rolling circle amplification step was used to subject to proximity ligation (<40 nm) and circularization of the DNA. After the amplification process, replications of the DNA circle were labelled by complementary oligonucleotide probes and the signals were observed under a confocal microscope (LSM 510; Carl Zeiss). Representative cells from three fields of view were selected and photographed. All images were of single focal planes.

### 2.20 Statistical analyses

Results are expressed as means ± SEM for at least three independent experiments. Data were analysed using a one‐way ANOVA, followed by Bonferroni's post hoc test for multiple comparisons (SPSS 20; SPSS).

## 3 RESULTS

### 3.1 GIT1 deficiency impairs angiogenesis in fracture callus tissue

**Figure 1:**
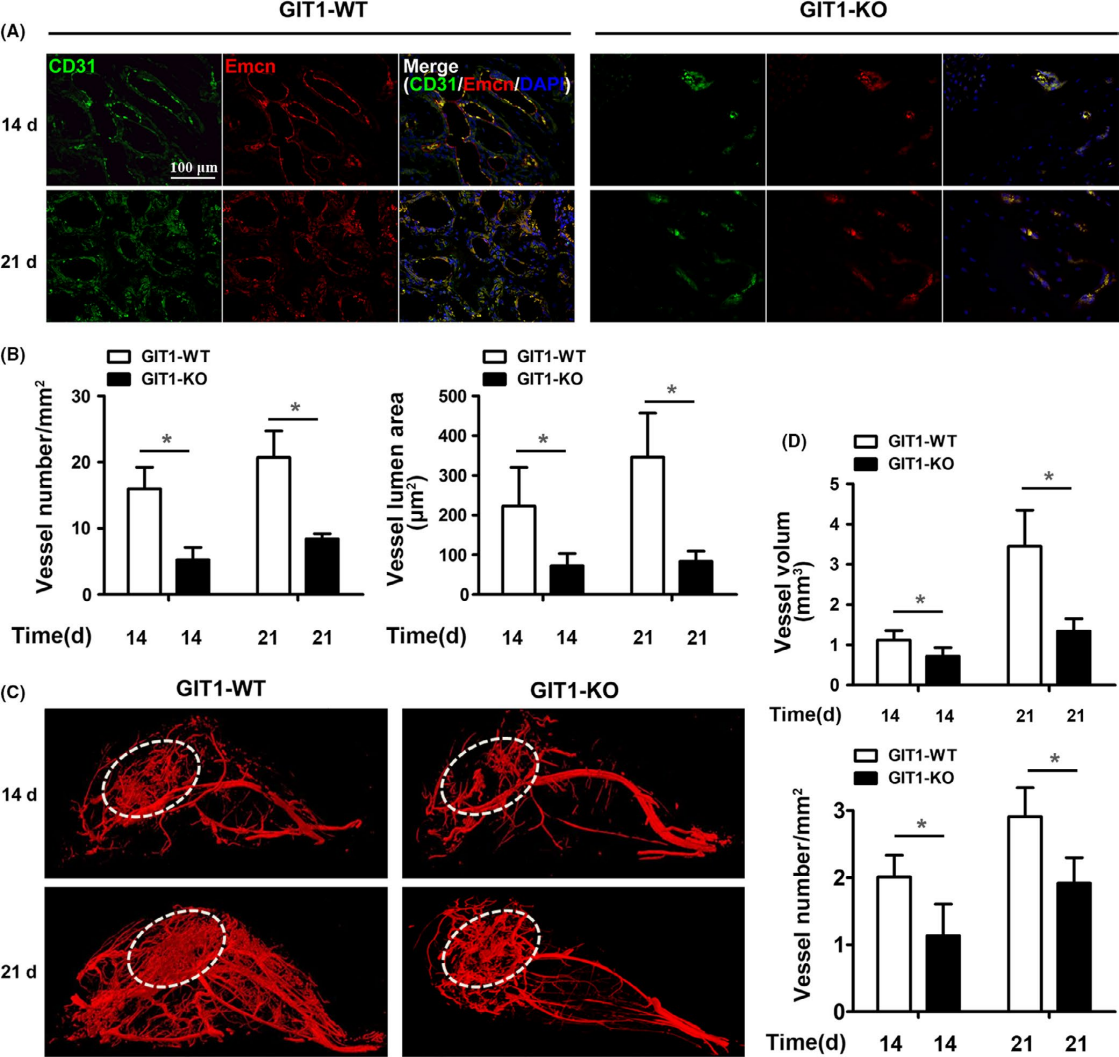
GIT1 deficiency inhibits angiogenesis in fracture callus tissue. A, Representative immunostaining images for CD31 (in green) and EMCN (in red) in the callus tissues of GIT1 WT and KO mice 14 and 21 days post‐fracture. Scale bar, 100 μm. B, Quantification of blood vessel number and lumen area in CD31^+^/ EMCN^+^ blood vessels (A) in the callus tissues. Data are represented as means ± SEM (n = 3 for both WT and KO mice). *P < .05. C, To visualize and quantify callus vascularity, WT and GIT1 KO mice were perfused with 4% PFA followed by MICROFIL® injection compounds. Representative images for vascular micro‐CT reconstructions of harvested femora 14 and 21 days post‐fracture. D, Quantification of callus vascular parameters, including vessel volume and vessel number. Data are represented as means ± SEM (n = 3 for both WT KO mice). *P < .05

GIT1 KO mice were previously generated to explore the putative effects and mechanisms of GIT1 on fracture repair.^34^, ^35^, ^37^, ^38^, ^40^ Unlike in WT mice, histology revealed greater volumes of cartilaginous callus still present on day 21 in the fracture callus tissue of GIT1 KO mice (Figure S1), consistent with results of our previous study.^38^, ^40^ Recent studies have revealed that a distinct capillary subtype known as type H, characterized by high expression of endothelial markers CD31 and endomucin (EMCN), couples angiogenesis and osteogenesis.^41^, ^42^ CD31 and EMCN immunofluorescence double staining was performed to verify the effects of GIT1 on effective angiogenesis for bone formation during fracture healing. Angiogenic vessels, co‐labelled with CD31 and EMCN and morphologically complicated with a larger lumen area, can be observed in the callus area of WT mice, but not in that of GIT1 KO mice 14 and 21 days post‐fracture (Figure 1A,B). Therefore, quantitative vascular micro‐CT analyses were performed to evaluate neovascularization 14 and 21 days post‐fracture in WT and GIT1 KO mice. Representative reconstructions indicated reduced callus vascularity in KO mice, compared to their WT littermates, with GIT1 KO mice displaying a marked reduction in vessel volume and number (Figure 1C,D). These results indicate that impaired angiogenesis may be an important factor resulting in delayed fracture union.

### 3.2 GIT1 deficiency inhibits expression and secretion of VEGF in BMSCs

**Figure 2:**
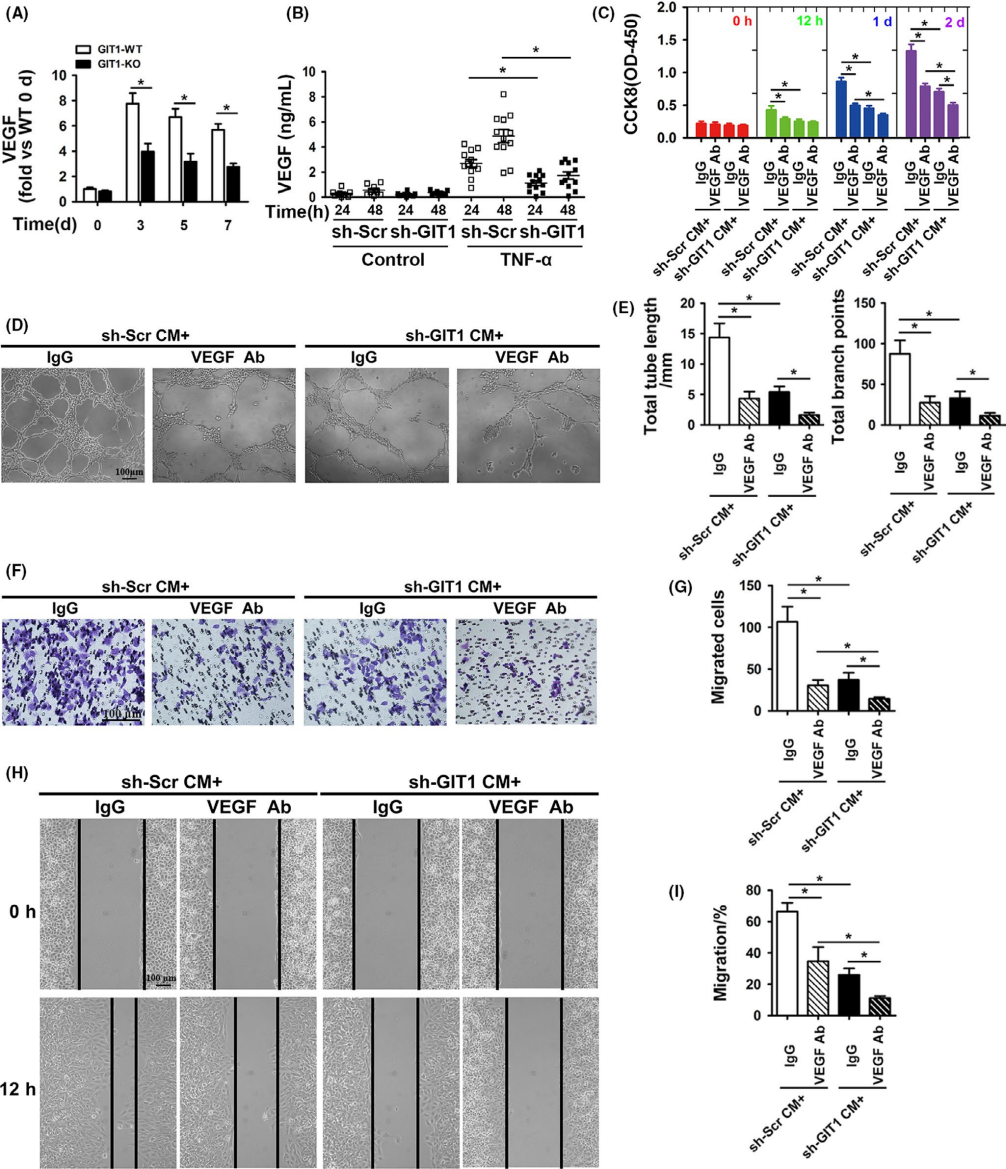
GIT1 deficiency inhibits expression and secretion of VEGF in BMSCs. (A) VEGF mRNA detected by qPCR in mBMSCs by direct adherent 24 hours culture from bone marrow adjacent to the fracture site in GIT1 WT and KO mice between 0 and 7 days post‐surgery. Data are represented as means ± SEM (n = 3 for both WT and KO mice). *P < .05. (B) hBMSCs were transfected with sh‐GIT1 or sh‐Scr for 48 hours and then subjected to control sham or TNF‐α (10 ng/mL) for 24 and 48 hours. VEGF concentration was detected by ELISA in hBMSC‐CM (n = 6). *P < .05. (C) Proliferation of HUVECs cultured with hBMSCs‐CM examined by CCK8 12 hours, 1, and 2 d after TNF‐α stimulation. Data are represented as means ± SEM (n = 8). *P < .05. (D, F, H) Representative images of Matrigel tube formation, transwell and scratch wound assays with hBMSCs‐CM cultures after TNF‐α stimulation for 48 hours. Scale bar, 100 μm. (E, G, I) Quantitative analysis of tube length and branch points during tube formation (D), the number of migrated cell (F) and migration rate of HUVECs (I). Data are represented as means ± SEM (n = 6). *P < .05

VEGF plays critical roles in bone repair, since angiogenesis and osteogenesis are often coupled. However, whether GIT1 KO affects the expression of VEGF in BMSCs has not been studied. First, mBMSCs were collected from bone marrow adjacent to the fracture site in GIT1 WT and KO mice between 0 and 7 days post‐fracture. The mBMSC phenotype was identified using morphological images and flow cytometry. The morphology of BMSCs was round, spindle, polygonal at P0 and became long fusiform at P3 (Figure S2A), characterized by BMSC surface markers (97.9% CD44, 2.91% CD45, 91.7% CD90 and 88.3% CD105, Figure S2B). VEGF mRNA expression in BMSCs was detected by qPCR and reached the highest levels 3 days post‐fracture, decreasing gradually thereafter. The level was lower in GIT1 KO than in WT at the same time post‐fracture (Figure 2A). Though, GIT1 also affected the expression of other angiogenic factor mRNAs, such as Ang‐1, FGF, HGF and TGF‐β, its effect on VEGF was most remarkable (Figure S3).

To further confirm the effects of GIT1 knockdown on secreting angiogenic factors, such as VEGF in hBMSCs, GIT1 was knocked down in hBMSCs using specific shRNAs. Knockdown efficiency was examined using Western blotting (Figure S4). First, VEGF concentrations after TNF‐α (10 ng/mL) stimulation for 24 and 48 hours detected by ELISA in the CM from normal hBMSCs were significantly increased compared to hBMSCs with GIT1 deletion, but not under physiological conditions (Figure 2B). CM from normal hBMSCs under TNF‐α stimulation for 48 hours induced significantly more HUVEC proliferation as shown by CCK8, as well as tube formation and migration evaluated using the Transwell and scratch wound assays of HUVECs vs. CM from GIT1‐knockdown‐BMSCs. Significant differences were still observed in proliferation or migration after treatment with a VEGF‐neutralizing antibody (Figure 2C‐I). Furthermore, GIT1 decreased relevant angiogenic factors in hBMSCs after the TNF‐α stimulation (Figure S5), which was consistent with results in vivo. These results suggest that VEGF is not the only factor, of which secretion is inhibited after GIT1 deficiency.

### 3.3 GIT1 KO inhibits activation of the Notch signal

**Figure 3:**
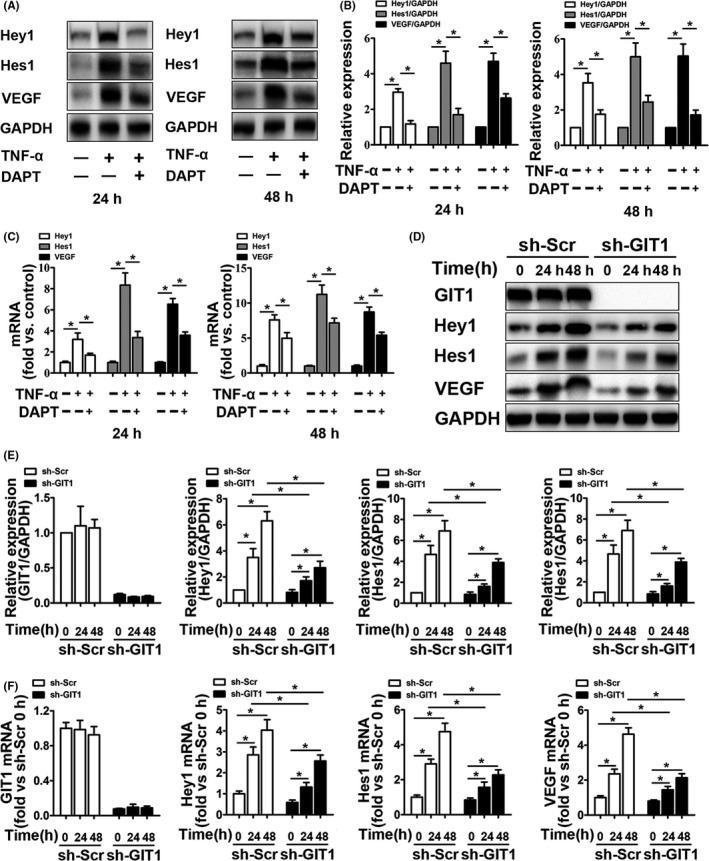
GIT1 KO inhibits activation of the Notch signal. A, Representative Western blots for Hey1, Hes1 and VEGF expression in TNF‐α‐treated (24 and 48 hours) hBMSCs in the presence or absence of Notch inhibitor DAPT. B, Quantification of Hey1, Hes1 and VEGF in hBMSCs based on Western blots described in (A). GAPDH was used as loading control. Data are represented as means ± SEM (n = 3). *P < .05. C, qPCR expression analysis for Hey1, Hes1, and VEGF mRNA in hBMSCs treated as described in (A). Data are represented as means ± SEM (n = 3). *P < .05. D, Representative Western blots for GIT1, Hey1, Hes1 and VEGF expression in hBMSCs transfected with sh‐GIT1 or sh‐Scr for 48 hours, subjected to control sham operation or TNF‐α stimulation for 24 and 48 hours. E, Quantification of GIT1, Hey1, Hes1, and VEGF in hBMSCs based on Western blots described in (D). GAPDH was used as loading control. Data are represented as means ± SEM (n = 3). *P < .05. F, qPCR expression analysis for Hey1, Hes1 and VEGF mRNA in hBMSCs treated as described in (D). Data are represented as means ± SEM (n = 3). *P < .05

Our previous study has shown that the Notch signal is involved in activation of the inflammation‐induced NF‐κB signal.^29^ Thus, we hypothesized that inhibition of the Notch signal can suppress the expression of VEGF in hBMSCs induced by TNF‐α. First, the Notch inhibitor DAPT decreased the expression of Hey1 and Hes1, the Notch signal targets, and VEGF in hBMSCs after the TNF‐α stimulation (Figure 3A–C and Figure S6). Additionally, the GIT1 knockdown significantly suppressed expression of Hey1, Hes1 and VEGF after TNF‐α stimulation. Thus, we believe that GIT1 may regulate VEGF expression by modulating the Notch signal.

### 3.4 GIT1 KO inhibits activation of canonical NF‐κB signal

**Figure 4:**
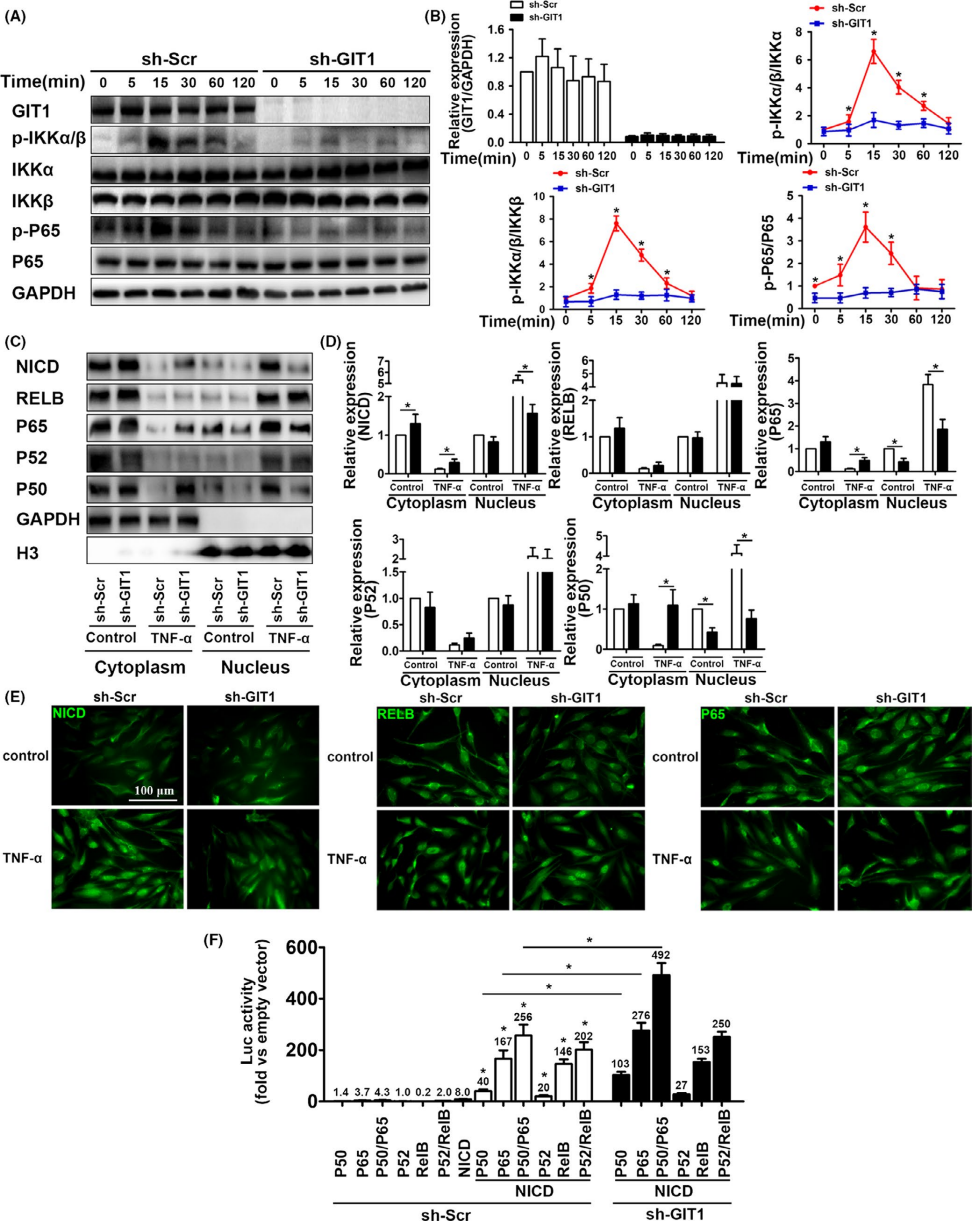
GIT1 KO inhibits activation of canonical NF‐κB signal. (A) Representative Western blots for total protein (GIT1, IKKα, IKKβ and P65) and phosphorylated protein (IKKα, IKKβ and P65) levels in GIT1 knockdown and control hBMSCs, exposed to TNF‐α stimulation for the indicated times. (B) Quantitative comparison of total protein and signalling activation levels between GIT1 knockdown and control hBMSCs using density scanning of the blots described in (A). GAPDH was used as loading control. Data are expressed as means ± SEM (n = 3). *P < .05 vs. hBMSCs infected with sh‐Scr. (C) Representative Western blots for cytoplasmic and nuclear protein (NICD, RELB, P65, P52 and P50) levels in GIT1 knockdown and control hBMSCs exposed to TNF‐α stimulation for 2 hours. (D) Quantification of cytoplasmic and nuclear protein (NICD, RELB, P65, P52, and P50) levels in hBMSCs based on Western blots described in (C). GAPDH and H3 were used as loading controls for cytoplasmic and nuclear proteins, respectively. Data are represented as means ± SEM (n = 3). (E) Representative immunostaining images for hBMSCs infected with sh‐GIT1 or sh‐Scr, treated with control sham operation or TNF‐α for 2 hours and co‐labelled for NICD, RELB, P65 (in green) and DAPI for subcellular co‐localization examination. Scale bar, 100 μm. (F) Reporter activity in HEK 293T cells co‐transfected with RBP‐jκ‐Luc and/or NICD‐, RELB‐, P65‐, P52‐ and P50‐expressing vectors. After 48‐hours transfection, luciferase activity was measured and fold increase vs. empty vector was calculated. Data are represented as means ± SEM (n = 5). *P < .05

Currently, cellular mechanisms by which GIT1 regulates the NF‐κB signal remain unclear. In vitro results showed that specific knockdown of GIT1 decreased IKKα/β and P65 phosphorylation, especially 15 minutes after TNF‐α treatment (Figure 4A,4). In addition, GIT1 expression in sh‐Scr‐transfected hBMSCs was unchanged 15‐120 minutes post‐treatment with TNF‐α or control sham operation (Figure 4A,B). Nuclear entry of NF‐κB subunits and NICD can activate the NF‐κB and Notch signals, respectively. Among the NF‐κB subunits, P65/P50 refers to the canonical NF‐κB signal, while RELB/P52 represents the non‐canonical signal.^19^, ^20^ Considering mutual regulation between the NF‐κB and Notch signals, expression of NF‐κB subunits and NICD in the cytoplasm and nucleus was detected using Western blotting. After 2 hours of TNF‐α stimulation, expression of the NF‐κB subunits and NICD in the nucleus increased significantly, while the corresponding protein in the cytoplasm decreased significantly (Figure 4C,D). Under TNF‐α stimulation, GIT1 knockdown in hBMSCs did not affect nuclear entry of RELB and P52, but significantly inhibited translocation into the nucleus of P65, P50 and NICD (Figure 4C,D). Subsequently, similar results were obtained by immunofluorescence analysis, which showed that GIT1 knockdown impaired nuclear localization of P65 and NICD, but not RELB (Figure 4E). To further examine whether GIT1 deletion affects the activation of Notch depending on the nuclear entry of P65/P50, RELB, P65, P52 and P50, were overexpressed in the presence or absence of low‐dose NOTCH‐NICD in HEK 293T cells with a RBP‐jκ‐Luc reporter construct, which contains six RBP‐jκ response elements in front of the luciferase gene (Figure 4F). Overexpression of RELB, P65, P52 and P50 alone or together had little effect on the luciferase activity, while low‐dose NICD increased the luciferase activity 8‐fold. Importantly, overexpression of RELB, P65, P52 and P50 markedly increased the NICD‐induced RBP‐jκ‐Luc activity, while RELB/P52 or P65/P50 combined had a synergistic effect. Furthermore, luciferase activity was higher when P65 and P50 were overexpressed together or separately in GIT1 deletion cells than in WT cells, but not when RELB and P52 were overexpressed together or separately. Thus, we believe that GIT1 directly modulates the canonical NF‐κB signal, leading to the activation of Notch.

### 3.5 SLD structure containing GIT1 CC2 domain plays a critical role in the interaction with NEMO CC2 domain

**Figure 5:**
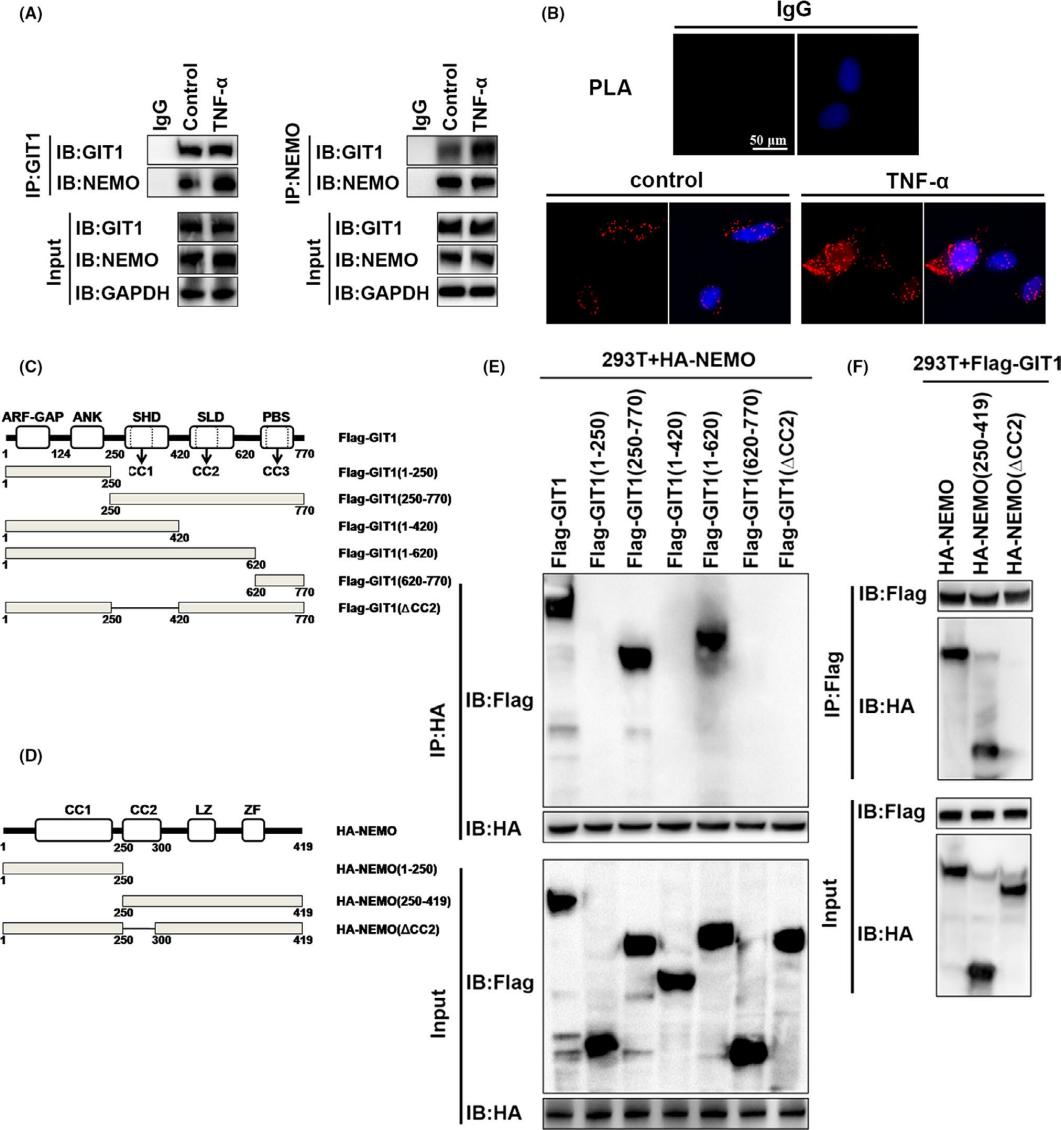
SLD structure containing GIT1 CC2 domain plays a critical role in interaction with NEMO CC2 domain. (A) Association of endogenous GIT1 and NEMO in hBMSCs without treatment or after TNF‐α stimulation for 2 hours, was examined by IP with GIT1 antibody, immunoblotting for NEMO or IP with NEMO antibody, and immunoblotting for GIT1. (B) Fixed and permeabilized hBMSCs from (A) were first incubated with rabbit NEMO and mouse GIT1 antibodies. Subsequently, cells were incubated with anti‐mouse MiNUs PLA probes followed by ligation and amplification. Interacting proteins were visualized using red fluorophore‐labelled oligonucleotides. Fixed and permeabilized cells were incubated with mouse IgG and rabbit IgG for use as controls. Cells were then incubated with anti‐mouse MiNUs PLA probes and visualized using red fluorophore‐labelled oligonucleotides, as above. (C, D) Functional domains of GIT1 and NEMO. (E) HEK293T cells were co‐transfected with Flag‐GIT1‐WT or Flag‐GIT1 deletion mutants and HA‐NEMO for 48 hours and subsequently treated with TNF‐α for 15 minutes. IP was performed with Flag antibody and probed for HA to detect interaction of NEMO and GIT1 or GIT1 deletion mutants. Cell lysates were also examined directly by immunoblot analysis with HA or Flag antibodies. (F) HEK293T cells were co‐transfected with HA‐NEMO‐WT or HA‐NEMO (ΔCC2) and Flag‐GTI1‐WT. Interaction of GIT1 and NEMO or NEMO (ΔCC2) was examined by IP with HA antibody and immunoblotting for Flag antibody. Cell lysates were examined directly by immunoblot analysis with antibodies. ANK, ankyrin‐rich repeat domain; ARF‐GAP, amino‐terminal ADP‐ribosylation factor‐GTPase‐activating protein domain; LZ, leucine zipper domain; TBD, thioredoxin binding region; ZF, zinc finger

**Figure 6:**
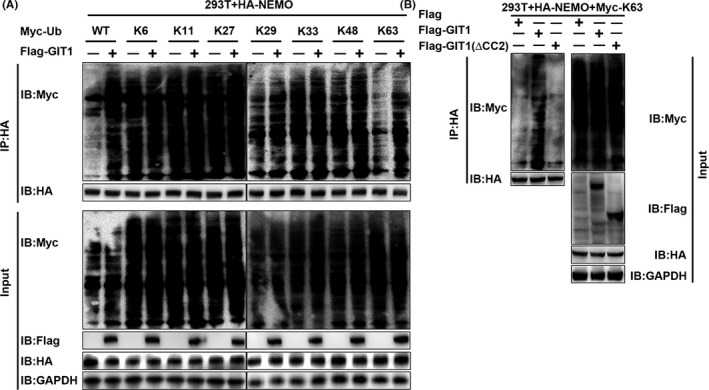
GIT1 enhances affinity of NEMO for K63‐linked ubiquitin chains via CC2 of GIT1. A, HEK293T cells were transfected with Fag‐GIT1‐WT, HA‐NEMO‐WT, Myc‐ubiquitin‐WT, and Myc‐K6‐, K11‐, K27‐, K29‐, K33‐, K48‐ or K63‐linked‐Ub for 48 hours and subsequently treated with TNF‐α for 2 hours following MG132 (10 μM) treatment for 1 hour. HA‐immunoprecipitation was performed and analysed using anti‐Myc antibody by immunoblot analysis. B, HEK293T cells were transfected with HA‐NEMO‐WT, Myc‐ and K63‐linked‐Ub, and Flag‐GIT1, or Flag‐GIT1(ΔCC2) for 48 hours. Harvested protein was treated with TNF‐α for 2 hours following MG132 (10 μM) treatment for 1 hour, immunoprecipitated with anti‐HA and immunoblotted with anti‐Myc

GIT1 contains three putative coiled‐coil (CC) domains, including CC1 (aa 254‐274), CC2 (aa 424‐474) and CC3 (aa 649‐669). Similar structures in NEMO are CC1 (aa 61‐195) and CC2 (aa 250‐300). Our previous studies have shown that GIT1 can interact with certain proteins, such as ASK1,^34^ ERK1/2,^43^, ^44^ and sorting nexin‐6,^45^ via similar CC structures between them. A recent study has reported that NEMO binds to the E3 ubiquitin ligase TRIM29, inducing NEMO degradation via the CC domain in TRIM29.^46^ Therefore, we predicted that GIT1 would bind to NEMO via the CC domain of each protein. First, co‐IP experiments and indirect in situ PLAs that visualize protein interactions using red fluorophore‐labelled oligonucleotides showed that endogenous NEMO interacted and co‐localized with GIT1 in hBMSCs under physiological conditions or in response to TNF‐α stimulation (Figure 5A,B). Interestingly, interaction between GIT1 and NEMO was slightly increased in TNF‐α‐treated cells. In contrast, NC, where mouse IgG and rabbit IgG were used, revealed negligible non‐specific binding of PLA probes (Figure 4B). To determine whether GIT1 interacts with NEMO via the CC2 domain, HA‐tagged full‐length NEMO, Flag‐tagged full‐length GIT1 and truncation mutants (Figure 6C) were co‐expressed in HEK293T cells. Result of the co‐IP demonstrated that GIT1 binding to NEMO was mediated by SLD structure that contained the CC2 domain (Figure 5E). To determine the interaction domain in NEMO, Flag‐tagged GIT1 was co‐expressed with HA‐tagged full‐length NEMO or mutants in HEK293T cells (Figure 6D). Full‐length GIT1 was strongly associated with full‐length NEMO, but weakly with NEMO (ΔCC2; Figure 6F). Collectively, these results highlight the interaction of NEMO with GIT1 via the CC2 domain.

### 3.6 GIT1 enhances NEMO affinity for K63‐linked ubiquitin chains via CC2 of GIT1

NEMO has two distinct ubiquitin‐binding domains (UBDs). One is composed of the CC2 and leucine zipper (LZ) domains, together called the UBAN domain, while the other is composed of the C‐terminal zinc finger (ZF) motif.^47^ It has been shown that NEMO binds to the polyubiquitin chains, such as K11‐linked and K63‐linked chains, via its UBDs for the NF‐κB activation.^21^, ^22^, ^23^, ^24^, ^25^ To investigate whether NEMO undergoes ubiquitination and which types of NEMO ubiquitination GIT1 could affect, HEK293T cells were transfected with plasmids expressing HA‐NEMO, and Myc‐(HA‐Ub (WT), K6‐linked‐Ub, K11‐linked‐Ub, K27‐linked‐Ub, K29‐linked‐Ub, K33‐linked‐Ub, K48‐linked‐Ub, or K63‐linked‐Ub), together with the empty vector or expression vector of Flag‐GIT1. Overexpression of GIT1 markedly increased WT and K63‐linked ubiquitination of NEMO, but had no appreciable effect on the ubiquitination of NEMO with other linkages (K6, K11, K27, K29, K33 or K48; Figure 6A). To study whether such ubiquitination of NEMO is dependent on the binding site at which GIT1 interacts with NEMO, we transfected HEK293T cells with plasmids expressing HA‐NEMO, Myc‐K63‐linked‐Ub, Flag‐full‐length GIT1 or mutant GIT1 (ΔCC2). Immunoblot analysis demonstrated that NEMO ubiquitination was substantially enhanced by overexpression of GIT1, not mutant GIT1 (ΔCC2, Figure 6B). Taken together, it is possible to conclude that the CC2 domain of GIT1 is essential for NEMO K63‐linked ubiquitination.

### 3.7 GIT1 does not affect K63‐linked ubiquitination of RIP1 induced by TRAF2

**Figure 7:**
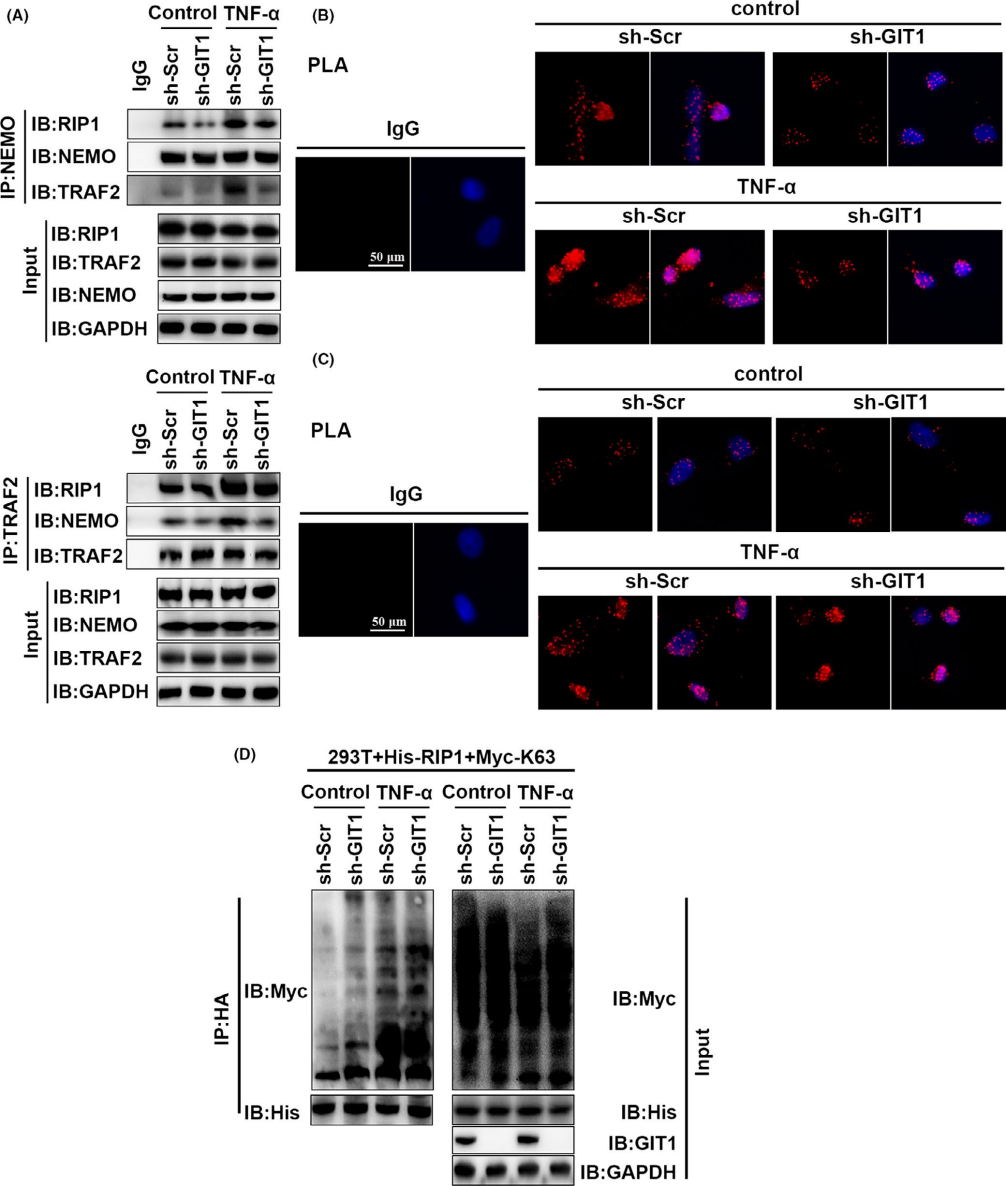
GIT1 does not affect K63‐linked RIP1 ubiquitination induced by TRAF2. A, Association of endogenous TRAF2 and NEMO in GIT1 knockdown and control hBMSCs without treatment or after TNF‐α stimulation for 2 hours was examined by IP with NEMO antibody, immunoblotting for TRAF2 and RIP1 or IP with TRAF2 antibody, and immunoblotting for NEMO and RIP1. B, C, Fixed and permeabilized hBMSCs treated as described in (A) were first incubated with rabbit NEMO antibody and mouse TRAF2 antibody (B) or mouse TRAF2 antibody and rabbit RIP1 antibody (C). Subsequently, cells were incubated with anti‐mouse MiNUs PLA probes followed by ligation and amplification. D, HEK293T cells were transfected with His‐RIP1‐WT, Myc‐ and K63‐linked‐Ub and sh‐GIT1, or sh‐Scr for 48 hours. Harvested protein was treated with TNF‐α for 2 hours following MG132 (10 μM) treatment for 1 hour, immunoprecipitated with anti‐His, and immunoblotted with anti‐Myc

TNF‐α can induce ubiquitination of the TRAF2 activation substrates,^29^, ^48^, ^49^ such as RIP1, leading to K63‐linked ubiquitination,^21^, ^22^, ^23^, ^24^, ^25^ thereby recruiting TAK1‐TAB2/TAB3^50^ and IKK complexes consisting of two kinase subunits IKKα and IKKβ and a regulatory subunit NEMO. The TAK1‐TAB2/TAB3 complex can subsequently trigger IKK phosphorylation and activation.^48^ To determine whether GIT1 is involved in the affinity of NEMO for K63‐linked ubiquitin chains by modulating the K63‐linked ubiquitination of RIP1, interactions of NEMO, TRAF2 and RIP1 were observed. Results of the co‐IP experiment and PLA showed that endogenous NEMO co‐immunoprecipitated with TRAF2 or RIP1 under physiological conditions or in response to TNF‐α stimulation in hBMSCs (Figure 7A‐C). However, interaction of NEMO and TRAF2 or NEMO and RIP1 was decreased in TNF‐α‐treated cells with GIT1 deletion, but not of TRAF2 and RIP1 (Figure 7A‐C). Second, to observe whether ubiquitination of RIP1 is affected by GIT1, HEK293T cells were transfected with lentivirus expressing specific shRNA against GIT1 sequence or scramble shRNA, as well as plasmids expressing His‐RIP1 and Myc‐K63‐linked‐Ub. Immunoblot analysis demonstrated that GIT1 shRNA did not inhibit the ubiquitination of RIP1 under TNF‐α (Figure 7D). These results indicated that GIT1 does not affect TRAF2‐induced ubiquitination of RIP1.

## 4 DISCUSSION

In the present study, we found that GIT1 KO reduced the expression and secretion of angiogenic factors (VEGF, Ang‐1, FGF, HGF and TGF‐β) in BMSCs. Indeed, MSCs and its secreted angiogenic mediators have been shown to induce angiogenesis and thereby enhancing tissue repair and functional outcomes in a variety of pathologies associated with insufficient angiogenesis.^51^, ^52^, ^53^ Moreover, VEGF functions not only as one of the most important regulators of vascular development and angiogenesis, it also plays critical roles in skeletal development,^54^ which is consistent with our qPCR results in vivo and vitro. Although several other angiogenic factors in the GIT1 KO group also showed different degrees of decline, the change in VEGF mRNA was most remarkable. And, when we added VEGF antibody to CM‐induced endothelial cells (EC), the tube formation and migrated cells were significantly decreased. Hence, these results indicated that VEGF plays a unique role in this pathological process. Recently, the NF‐кB signal induced by inflammatory factors, such as TNF‐α, has been reported to crosstalk with the Notch pathway. For example, inflammatory factors regulate Notch signalling in several cell types using different mechanisms. These factors regulate expression of the Notch ligands and receptors (Notch 1‐4) in fibroblasts, endothelial cells and skeletal muscle cells,^55^, ^56^, ^57^ and activate Notch after NICD release by promoting the binding of NICD to RBP‐jк.^20^ Notch interactions with NF‐кB have mainly focused on the canonical NF‐кB subunits P65 and P50^58^ and NF‐кB transcription.^58^, ^59^, ^60^ Downregulation of Notch decreased the binding of NF‐κB subunits to their target gene promoter, reduced the NF‐κB expression and enhanced the inhibitory protein expression.^61^ Our previous study has shown that DAPT can suppress the NF‐кB signal by reducing NICD cleavage from the Notch molecules and combination between NICD and P65.^29^ In addition, Notch and NF‐кB signals together regulate the expression of VEGF in a variety of cells.^27^, ^62^, ^63^, ^64^, ^65^ Our study confirms that inhibition of the Notch pathway can inhibit the expression and secretion of inflammatory‐induced VEGF in BMSCs. Moreover, GIT1 KO inhibits activation of the Notch and NF‐кB signals under TNF‐α stimulation. Thus, we speculate that GIT1 affects the expression of VEGF by regulating the Notch and NF‐кB signals.

The mechanism by which GIT1 regulates the Notch and NF‐кB signals has not yet been reported. Our data demonstrated that GIT1 KO significantly inhibited the nuclear import of NICD and P65/P50, rather than RELB/P52. Considering that canonical and non‐canonical NF‐κB subunits associate with NICD and promote transportation of the latter into the nucleus,^20^, ^29^ we hypothesized that GIT1 specificity affects the canonical NF‐κB signal working upstream of the Notch signal in BMSCs. Further research shows that overexpression of RELB, P65, P52 and P50 did not affect the RBP‐jκ‐Luc reporter activity, whereas the NF‐κB subunits in combination with NICD significantly increased the luciferase activity expression. This indicates that NF‐κB subunits need NICD to regulate the Notch target gene expression. However, the NICD‐induced RBP‐jκ‐Luc activity was higher in GIT1 deletion cells than in the WT cells when overexpressing P65 and P50 alone or together, but not when overexpressing RELB and P52 alone or together. These results implied that GIT1 directly regulates the canonical NF‐κB signal as an upstream factor attributed to the activation of Notch.

In previous studies, we have shown that GIT1 contains three CC domains,^43^ the structure of which is similar to the CC domain of NEMO, which mediates NEMO trimerization.^66^, ^67^ Interaction between GIT1 and NEMO was demonstrated in BMSCs under physiological condition or inflammation stimulation and was slightly stronger in the latter. It was also shown that GIT1 interacted with NEMO via CC2 domains of GIT1 and NEMO. NEMO functions as a K63‐linked ubiquitin chain reader to mediate the downstream signalling cascade.^24^, ^25^ In the TNF‐α pathway, E3‐ligases belonging to the TRAF2 and cIAP1/2 are responsible for the K63‐linked ubiquitination of RIP1.^29^, ^48^, ^49^ K63‐ubiquitinated RIP1 is believed to be responsible for the recruitment of TAK1‐TAB2/TAB3 and IKK complex via TAB2^68^, ^69^ and NEMO, respectively,^23^, ^70^ allowing for TAK1‐independent trans‐autophosphorylation and activation of IKKβ in the IKK complex.^24^ In this study, we found a significant decline in the affinity of NEMO for K63‐linked ubiquitin chains by down‐regulating GIT1, but not the TRFA2‐induced K63‐linked ubiquitination of RIP1. Consistent with our data on the affinity of NEMO for the ubiquitin chains, we also observed that activation level of the IKK complex was reduced after the GIT1 knockdown. Based on our results, GIT1 regulates activation of the canonical NF‐κB signal by influencing the combination of NEMO and K63‐linked ubiquitination of RIP1 mediated by TRAF2.

**Figure 8:**
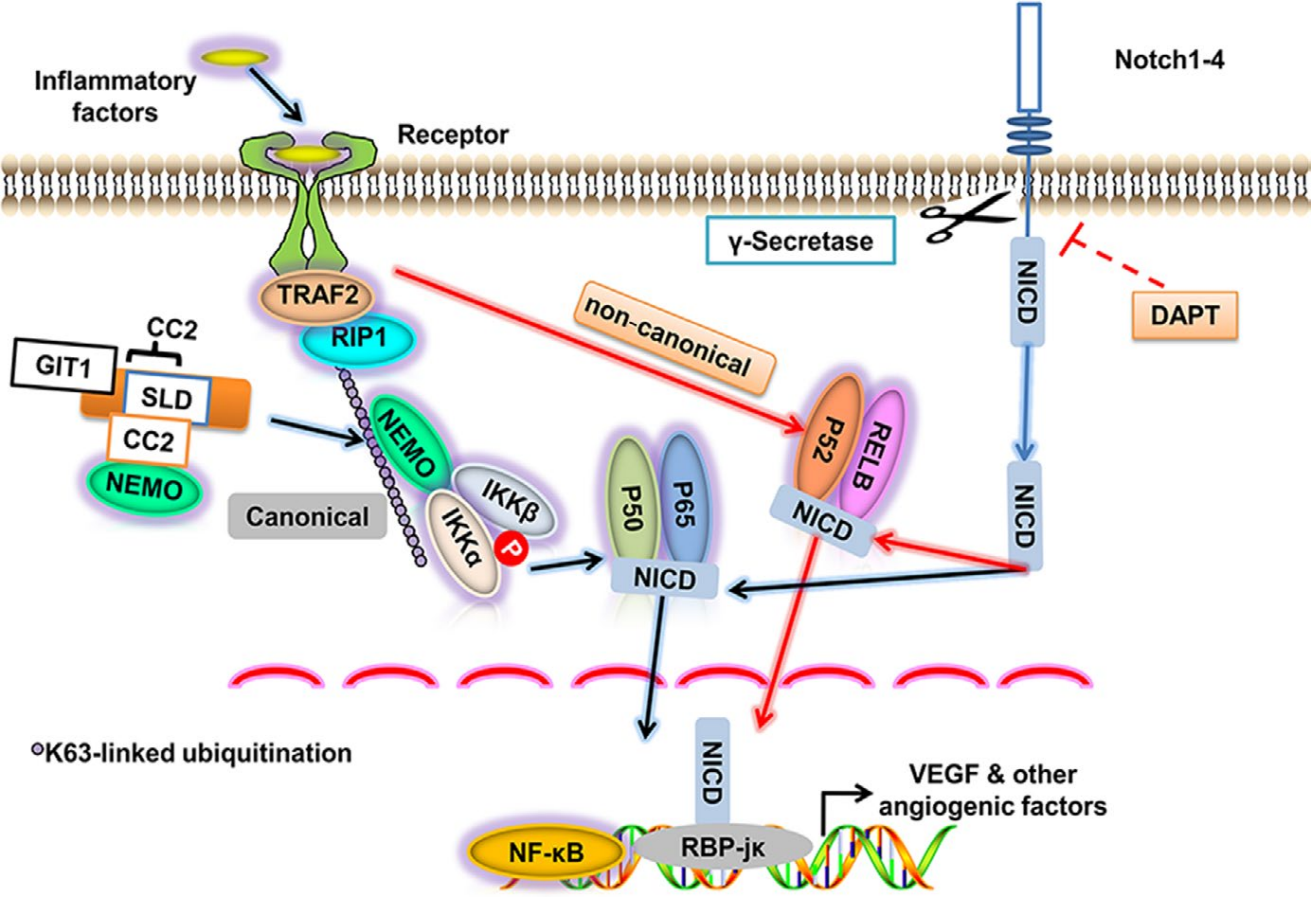
Working model of positive NF‐κB/Notch signal regulation by GIT1. GIT1 interacts with NEMO via their mutual CC2 structures and enhances NEMO affinity for K63‐linked ubiquitination of RIP1 induced by TRAF2 (Figure 8). It leads to phosphorylation of the IKKα/β/NEMO complex and facilitates activation of the canonical NF‐κB signal, but not the non‐canonical signal. It regulates the Notch signalling by increasing translocation of NICD into the nucleus and subsequently leading to interaction of NICD and RBP‐jк, requiring cleavage from the Notch molecules to be induced by γ‐secretase and blocked by DAPT. Therefore, endogenous GIT1 enhances nuclear import of the canonical NF‐κB subunits P65 and P50 and NICD, leading expression of the downstream target genes of the NF‐κB/Notch signal, such as VEGF and other angiogenic factors in BMSCs, during the early stages of fracture healing

Overall, the results of this study suggest that GIT1 promotes the expression of VEGF by affecting activation of the NF‐κB/Notch signalling pathway in the early stages of fracture as schematically represented in Figure 8, thereby regulating the process of vascular formation and fracture healing in the epiphyseal region. Verifying the role of GIT1 in BMSCs will not only expand our understanding of the mechanism of fracture healing, but also enrich the biological function of GIT1.

## CONFLICT OF INTERESTS

None of the authors have a conflict of interest to declare.

## AUTHOR CONTRIBUTION

Guoyong Yin, Jin Fan and Jian Chen designed the experiments. Linwei Li, Pengyu Tang, Zheng Zhou, Qian Wang and Tao Xu performed the experiments. Shujie Zhao, Yifan Huang, Fanqi Kong and Wei Liu analysed the data. Linwei Li and Dingfei Qian wrote the manuscript. Lin Cheng, Zhimin Zhou, Xuan Zhao was responsible for technical developments. Jian Chen and Gaojian Tao supervised the study procedure. Changjiang Gu, Yongjun Luo and Guoyong Yin critically revised the manuscript.

## Data Availability

The data that support the findings of this study are available from the corresponding author upon reasonable request.
