# Supplementary figures and images for: GIT1 regulates angiogenic factor secretion in bone marrow mesenchymal stem cells via NF‐κB/Notch signalling to promote angiogenesis
Source: Cell Prolif. 2019 Sep 10;52(6):e12689. doi: 10.1111/cpr.12689 (PMC6869488; doi:10.1111/cpr.12689)

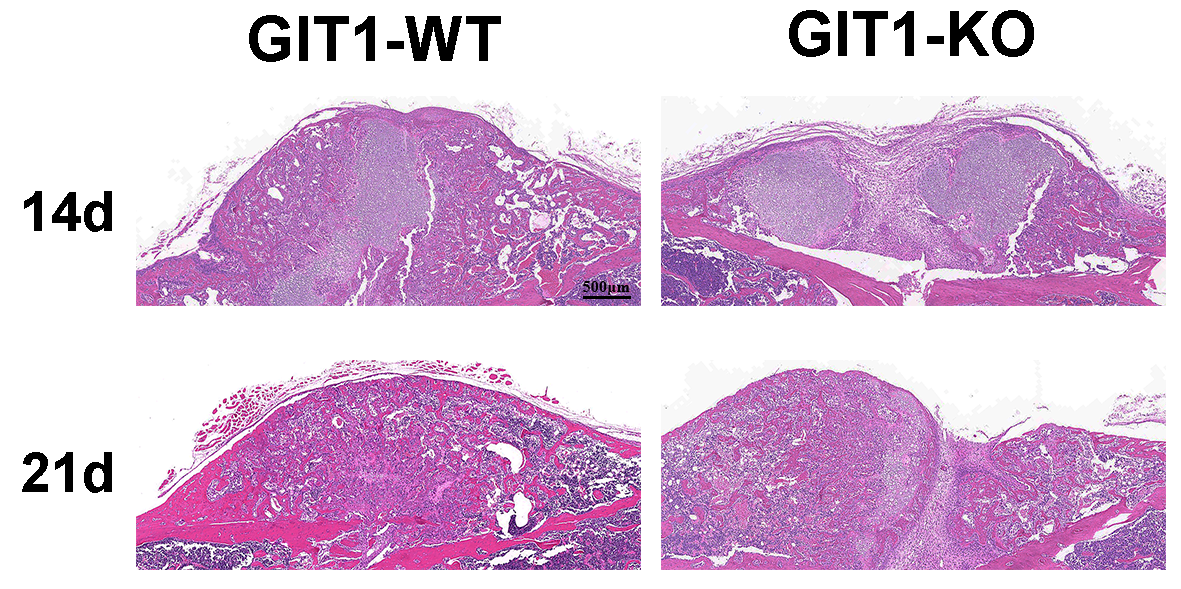

Supplement: Supplementary file 1 [file CPR-52-e12689-s001.tif]

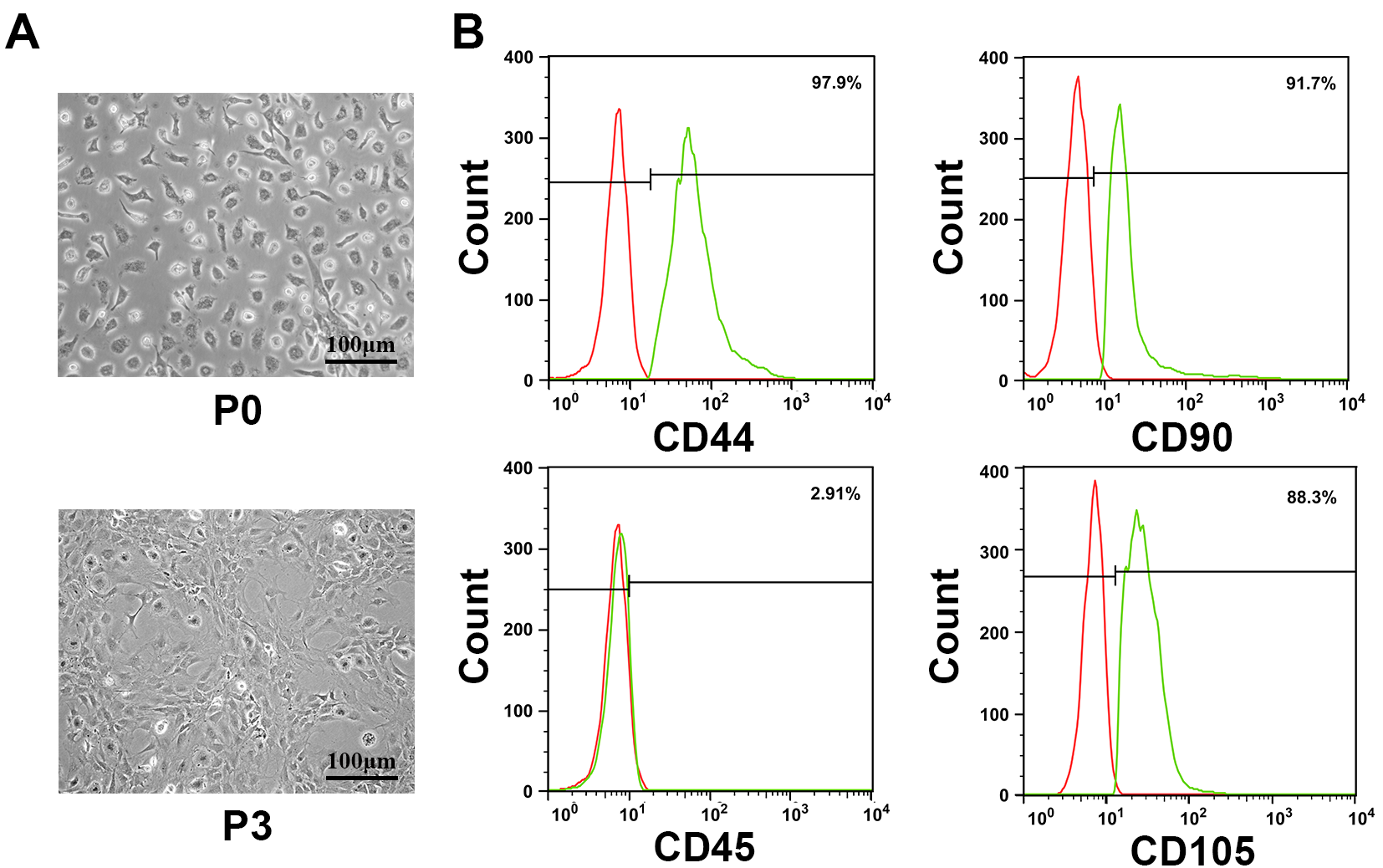

Supplement: Supplementary file 2 [file CPR-52-e12689-s002.tif]

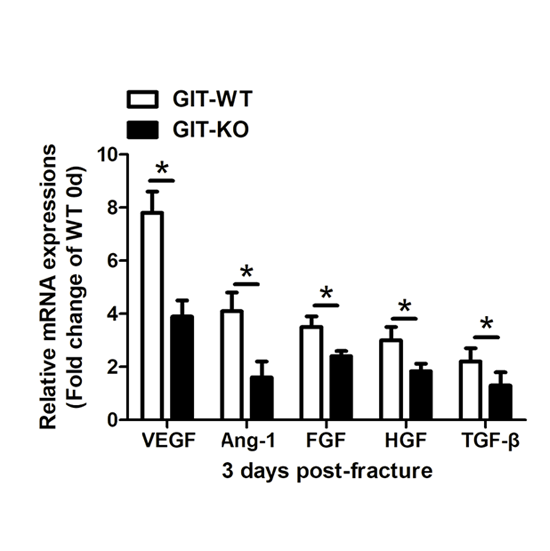

Supplement: Supplementary file 3 [file CPR-52-e12689-s003.tif]

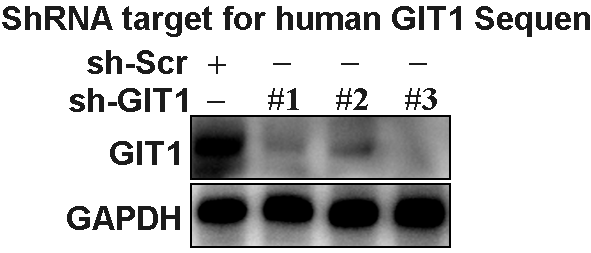

Supplement: Supplementary file 4 [file CPR-52-e12689-s004.tif]

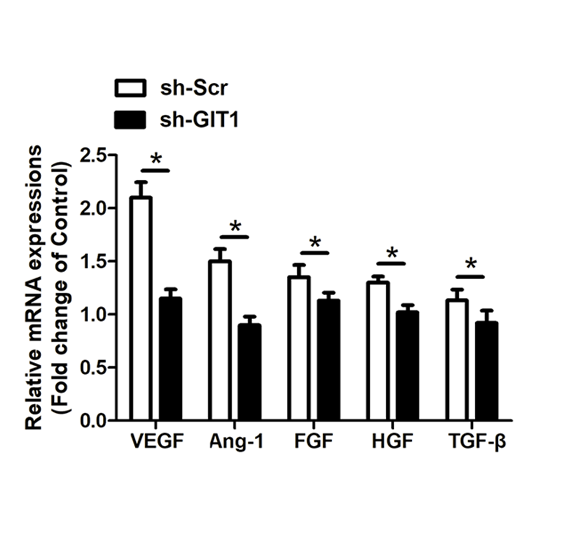

Supplement: Supplementary file 5 [file CPR-52-e12689-s005.tif]

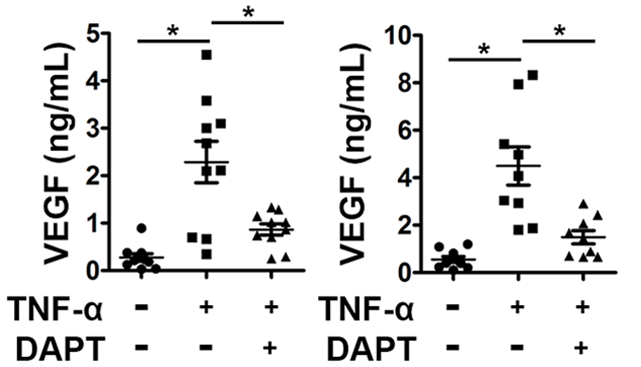

Supplement: Supplementary file 6 [file CPR-52-e12689-s006.tif]
