# Supplementary material for: GIT1 regulates angiogenic factor secretion in bone marrow mesenchymal stem cells via NF‐κB/Notch signalling to promote angiogenesis
Source: Cell Prolif. 2019 Sep 10;52(6):e12689. doi: 10.1111/cpr.12689 (PMC6869488; doi:10.1111/cpr.12689)
Supplement: Supplementary file 7 [file CPR-52-e12689-s007.doc]

**Supplemental Figure 1.** Histological analysis of fracture callus tissue was performed via H&E staining. Representative stains for calluses from WT and GIT1 KO mice 14 and 21 days post-fracture. (n = 3 for both WT and KO mice).

**Supplemental Figure 2.** Identification of adherent cells derived from bone marrow. mBMSCs by direct adherent culture for 24 h from bone marrow adjacent to the fracture site in GIT1 KO mice and littermates 0 to 7 days post-surgery. (n = 3 for both WT and KO mice). (A) Bright-field images showing morphologic character of P0 and P3 BMSCs. Scale bar, 100 μm. (B) P3 BMSCs were positive for CD44 (97.9%), CD45 (2.91%), CD90 (91.7%), and CD105 (88.3%), as analyzed by flow cytometry.

**Supplemental Figure 3.** Angiogenic factors mRNA detected by qPCR in mBMSCs by direct adherent 24 h culture from bone marrow adjacent to the fracture site in GIT1 WT and KO mice at 3 days post-fracture. Data are represented as means ± SEM (n = 3 for both WT and KO mice). *P<0.05.

**Supplemental Figure 4.** Knockdown efficiency of GIT1-specifc shRNA targeted for human sequences of GIT1 gene. Western blots for GIT1 expression knockdown in hBMSCs transfected with lentiviral vector carrying sh-GIT1 or sh-Scr. GAPDH was used as loading control. (n = 3).

**Supplemental Figure 5. H**BMSCs were transfected with sh-GIT1 or sh-Scr and then subjected to control sham or TNF-α (10 ng/mL) for 48 h. Angiogenic factors mRNA were detected by qPCR. Data are represented as means ± SEM (n =3). *P<0.05.

**Supplemental Figure 6.** VEGF concentration was detected by ELISA in hBMSC-conditioned medium. hBMSCs were subjected to control sham, TNF-α (10 ng/mL), or TNF-α + DAPT (10 μmol/mL) for 24 and 48 h. (CM) (n = 6). *P<0.05.
